# Year-round monitoring reveals prevalence of fatal bird-window collisions at the Virginia Tech Corporate Research Center

**DOI:** 10.7717/peerj.4562

**Published:** 2018-04-04

**Authors:** Rebecca M. Schneider, Christine M. Barton, Keith W. Zirkle, Caitlin F. Greene, Kara B. Newman

**Affiliations:** 1 Conservation Management Institute, Virginia Polytechnic Institute and State University (Virginia Tech), Blacksburg, VA, United States of America; 2 Virginia Polytechnic Institute and State University (Virginia Tech), Blacksburg, VA, United States of America; 3 Department of Biostatistics, Virginia Commonwealth University, Richmond, VA, United States of America; 4 Department of Fish and Wildlife Conservation, Virginia Polytechnic Institute and State University (Virginia Tech), Blacksburg, VA, United States of America

**Keywords:** Windows, Collisions, Bird conservation, Birds, Advocacy

## Abstract

Collisions with glass are a serious threat to avian life and are estimated to kill hundreds of millions of birds per year in the United States. We monitored 22 buildings at the Virginia Tech Corporate Research Center (VTCRC) in Blacksburg, Virginia, for collision fatalities from October 2013 through May 2015 and explored possible effects exerted by glass area and surrounding land cover on avian mortality. We documented 240 individuals representing 55 identifiable species that died due to collisions with windows at the VTCRC. The relative risk of fatal collisions at all buildings over the study period were estimated using a Bayesian hierarchical zero-inflated Poisson model adjusting for percentage of tree and lawn cover within 50 m of buildings, as well as for glass area. We found significant relationships between fatalities and surrounding lawn area (relative risk: 0.96, 95% credible interval: 0.93, 0.98) as well as glass area on buildings (RR: 1.30, 95% CI [1.05–1.65]). The model also found a moderately significant relationship between fatal collisions and the percent land cover of ornamental trees surrounding buildings (RR = 1.02, 95% CI [1.00–1.05]). Every building surveyed had at least one recorded collision death. Our findings indicate that birds collide with VTCRC windows during the summer breeding season in addition to spring and fall migration. The Ruby-throated Hummingbird (*Archilochus colubris*) was the most common window collision species and accounted for 10% of deaths. Though research has identified various correlates with fatal bird-window collisions, such studies rarely culminate in mitigation. We hope our study brings attention, and ultimately action, to address this significant threat to birds at the VTCRC and elsewhere.

## Introduction

In the United States, glass is responsible for an estimated 365–988 million bird deaths per year (Klem, 1990; Klem, 2009; Loss et al., 2014). In considering direct anthropogenic impacts to avian life in the US, the number of birds killed by glass is second only to the number killed each year (1.3–4.0 billion) by free-ranging domestic cats (Loss, Will & Marra, 2013). The literature has well documented the hazards of glass to birds (Banks, 1976; Klem, 1979; Klem, 1989; Klem, 1990; Dunn, 1993; Blem & Willis, 1998; O’Connell, 2001; Drewitt & Langston, 2008; Hager et al., 2008; Bayne, Scobie & Rawson-Clark, 2012; Machtans, Wedeles & Bayne, 2013; Hager & Craig, 2014; Kummer, Bayne & Machtans, 2016; Barton, Riding & Loss, 2017). However, studies of bird-window collision fatalities rarely lead to mitigating hazards.

Prior research has revealed several factors that influence avian mortality at windows and suggests that collisions can result from the interaction of these variables. Bird strikes are shown to increase in spring and fall when many birds are migrating (Borden et al., 2010; Ocampo-Peñuela et al., 2016), though year-round data is scant (Kummer, Bayne & Machtans, 2016). Building characteristics, such as the amount of glass, affect the likelihood that a collision will occur (Collins & Horn, 2008; Hager et al., 2013). The majority of fatal collisions in the US occur at low-rise buildings (Loss et al., 2014) and the positive correlation between building size and mortality is particularly strong in regions of low urbanization (Hager et al., 2017). Landscape features around buildings can affect collision rate, with extensive vegetation drawing birds in and reflecting in windows (Klem, 2009; Hager et al., 2008). Greenery mirrored by windows presents a false image of suitable habitat to birds that fly towards it, unable to distinguish the image from reality. Nocturnal light emitted by buildings attracts and disorients migrating birds (Ogden, 1996) and collisions typically peak during the morning and early afternoon hours (Klem, 1989; Kahle, Flannery & Dumbacher, 2016). Particularly vulnerable taxonomic families include Parulidae, Turdidae, and Emberizidae and long-distant migrants are generally at greater risk (Arnold & Zink, 2011). At the species level, disproportionate colliders include Ruby-throated Hummingbird, Black-throated Blue Warbler (*Setophaga caerulescens*), Ovenbird (*Seiurus aurocapilla*) Brown Creeper (*Certhia Americana*), Swamp Sparrow (*Melospiza georgiana*), and White-throated Sparrow (*Zonotrichia albicollis*) (Ogden, 1996; Hager et al., 2008; Borden et al., 2010; Loss et al., 2014).

Despite the recent research and attention documenting bird-window collisions at Duke University (Ocampo-Peñuela et al., 2016) and the Minnesota Vikings Stadium (Grant, 2014), building managers are often reluctant to take precautionary measures to reduce the threat of windows and glass to birds. Various deterrent materials are available for making windows and glass bird-friendly (e.g., window films, UV liquid, hanging cords), but large-scale implementation is lacking. Studies that evaluate the effectiveness of these products on a large scale are needed. Current legislation aimed at reducing bird-window collisions is insufficient. Only one state, Minnesota, and a few cities across the US, have mandated building standards for preventing bird strikes (American Bird Conservancy, 2012). The Federal Bird-Safe Buildings Act (H.R. 2280), introduced in May 2015, would require bird-friendly design standards on buildings newly constructed, acquired, or substantially renovated, and if passed would apply only to public buildings.

This study was initiated after a bird flew into the office window of R Schneider at the Virginia Tech Corporate Research Center (VTCRC) in mid-September 2013. VTCRC management granted permission to carry out the study and surveys began on October 19, 2013. The purpose of our research was to investigate all buildings at the VTCRC to determine the extent of bird-window collisions and examine possible effects of season, building characteristics, and surrounding landscape. We expected to observe an increase in fatal collisions at buildings with a greater surface area of glass, near extensive vegetation, and during spring and fall migration. Surveys were conducted year-round due to the lack of data gathered outside of the spring and fall migration (C Sheppard, pers. comm., 2013). If particularly hazardous buildings at the VTCRC were identified, we sought to apply and assess the effectiveness of collision deterrents on windows.

## Methods

### Study area

**Figure 1:**
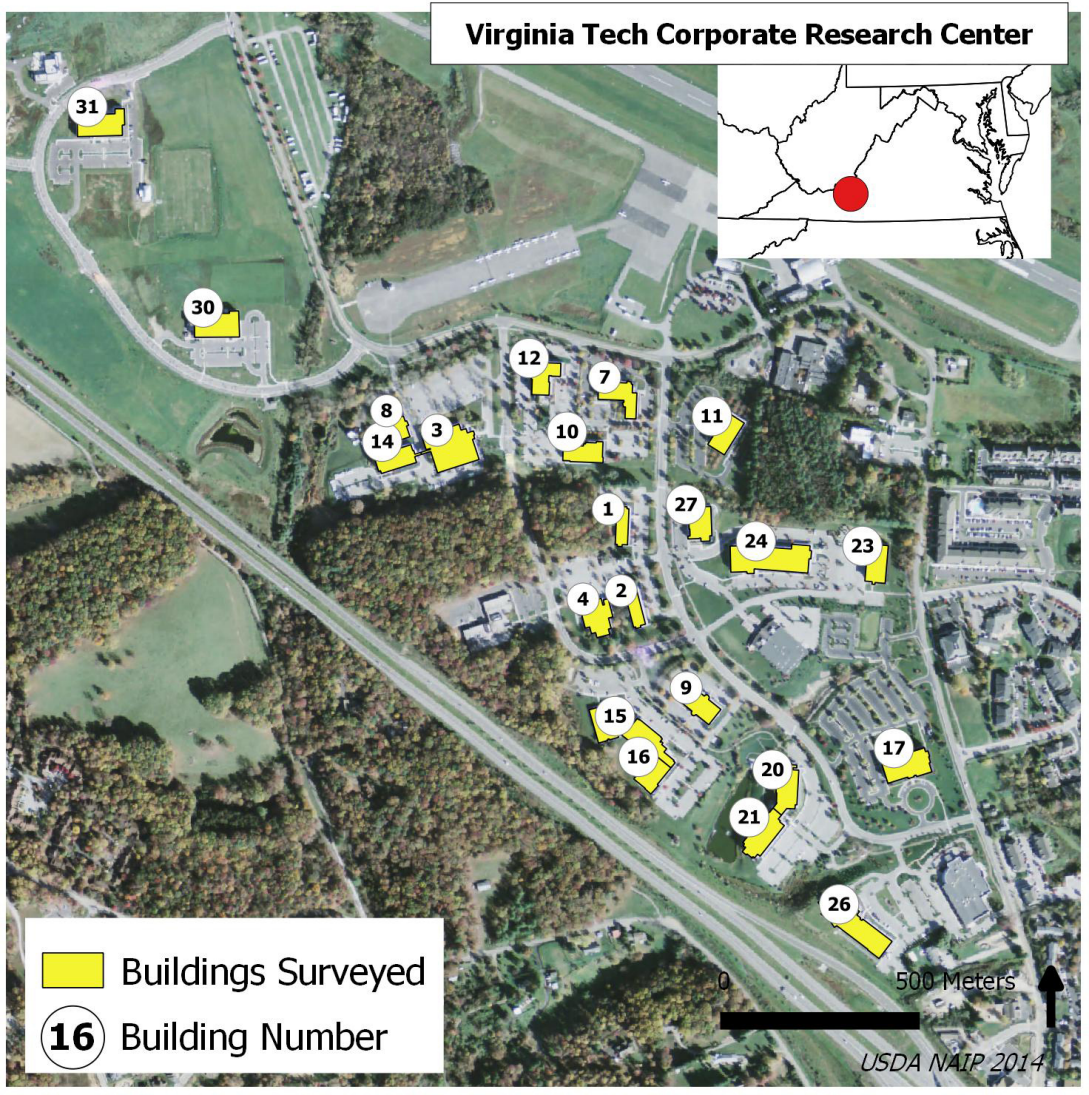
VTCRC map. Buildings surveyed for bird-window collisions at the VTCRC, Blacksburg, VA. Background image source USDA NAIP 2014.

**Figure 2:**
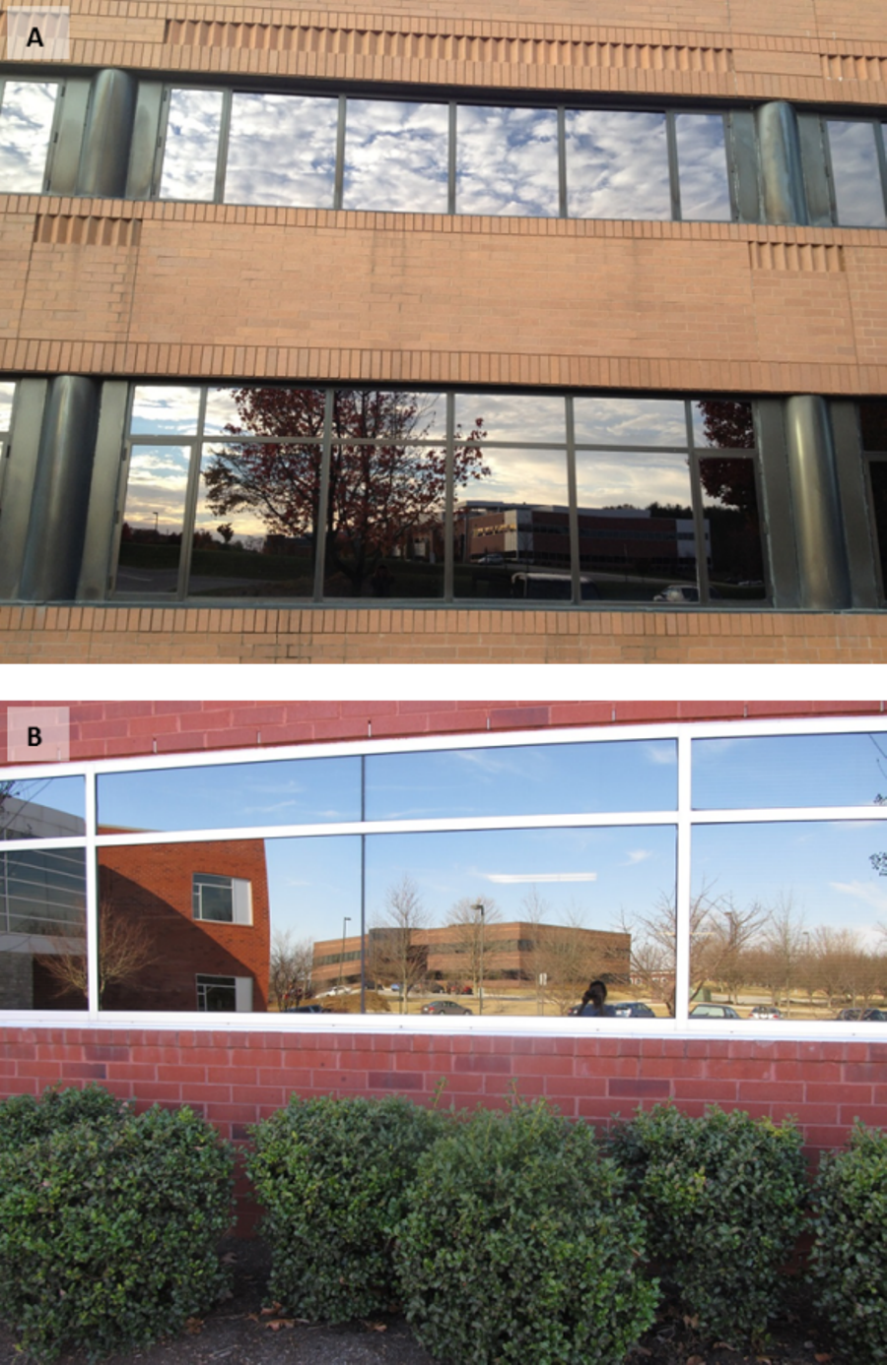
VTCRC windows. Typical highly reflective windows at the VTCRC, Blacksburg, VA (A) Building 2 (B) Building 15.

The VTCRC is located in Blacksburg, Virginia, United States (Fig. 1). At the time of the study, the office park consisted of 28 buildings on approximately 230 acres. The first building was constructed in 1988 and future plans include the construction of an additional 16 buildings (VTCRC, 2016). Buildings were assigned identification numbers that correspond with a pre-existing VTCRC building map (VTCRC, 2016). The majority of the buildings are two-story and only two buildings in the office park (buildings 12 and 17) have three stories. There is one glass walkway connecting buildings 3 and 14. Two man-made ponds are located in the VTCRC, the largest of which is located adjacent to buildings 20 and 21. Scattered ornamental trees are present throughout the office park, with larger forested patches on the western boundary and northeast corner of the property. Highly reflective and mirror-like windows comprise the majority of windows at the VTCRC (Fig. 2). Twenty-two buildings were monitored when schedules and weather allowed. The remaining six buildings were not surveyed due to ownership, or because the type of work performed in the buildings prohibited surveys.

### Collision surveys

We conducted year-round surveys opportunistically from October 19, 2013 until May 27, 2015. Methods were adapted from Hager & Cosentino (2014a) and Hager & Cosentino (2014b). A group of volunteers, comprised mostly of undergraduate students from Virginia Tech, carried out the surveys documenting all evidence of bird-window collisions. No carcasses were removed or collected; only photos were taken to document evidence of bird-window collisions (e.g., carcasses, feather piles). Each surveyor made one pass, walking slowly, around the perimeter of each building. The search area around each building was the width of the surveyor’s arms (approximately 2 m) held out horizontally from the building wall. Common substrates surveyed included lawn, pavement, and mulch. Surveyors also searched on top, behind, and inside of the shrubs next to the buildings. In addition to species information, date, start survey time, end survey time, building number, and building side were recorded. Surveys were not conducted during a specific time of day or in adverse weather conditions. Data was reported by volunteers every day or at minimum, once a week. We compared all findings, by photo or in the field, to previous findings to avoid double counting.

### Data measurement and analysis

Land cover was classified within a 50 m buffer for each of the 22 buildings surveyed. Virginia Geographic Information Network (VGIN) data was used to classify surrounding land cover to the following classes: forest, lawn (including pasture and turfgrass), impervious, water, and tree. To better classify scattered ornamental trees present at the VTCRC, the ‘tree’ land cover class was used to distinguish between sparse tree coverage and forest. We estimated glass area for each building using the program ImageJ (Rasband, 2016) following methods detailed by Hager & Cosentino (2014b). Glass area was centered at 0 and scaled when we used it our Bayesian regression model (Supplemental Information). Meteorological seasons were used to report seasonality of our results. Fall was defined as September–November, winter as December–February, spring as March–May, and summer as June–August.

Linear regression analyses were performed in the program R Core Team (2017), with the RStudio Team (2015), to explore possible relationships between the number of bird deaths per building and predictor variables, glass area and surrounding land cover. We relied on negative binomial regression for this analysis because Poisson distribution led to overdispersion in the data. In order to compare the number of bird deaths across buildings despite uneven survey effort, Poisson regression was performed with an offset for the number of observations. These results informed the Bayesian hierarchical regression model we used to estimate the relative risk of fatal collisions at the 22 buildings across the 20 months. We assumed a zero-inflated Poisson (ZIP) distribution for the number of fatal collisions observed at each building within each month. A ZIP model is common for handling excess zero counts in data and assumes these zeroes are generated by a process separate than the count values (Lambert, 1992). The model contains two components, a binomial distribution for the excess zeroes and a Poisson distribution for the count values (which may contain zeroes). The Poisson distribution assumes a mean of the product of the relative risk *θ*_*it*_ and the expected count *e*_*it*_ at building *i* in month *t*. The expected counts are the product of the overall fatal collision rate *r*_*t*_ in each month and the number of site observations at each building in each month.

We modeled the log of the relative risk *θ*_*it*_ for building *i* at month *t* as:

(1) \begin{eqnarray*}& & \log \nolimits \left( {\theta }_{it} \right) ={\beta }_{1}\times {\mathrm{PercentTree}}_{i}+{\beta }_{2}\times {\mathrm{PercentLawn}}_{i}+{\beta }_{3}\times {\mathrm{GlassArea}}_{i}+{\delta }_{t}\end{eqnarray*}

where *δ*_*t*_ is a temporal random effect. The parameter *β*_1_ is the log relative risk associated with a percent increase in the tree area within 50 m of a building and, similarly, *β*_2_ is the log relative risk associated with a percent increase in lawn area while *β*_3_ is the effect of a standard deviation increase in glass area on a building (SD = 175.63 m^2^). We chose these variables based on known relationships and significant relationships found in the exploratory analyses. The temporal term *δ*_*t*_ captured variation in the collisions across time not explained by the variables.

For the binomial distribution, we specified the probability of excess zeroes at building *i* as:

(2) \begin{eqnarray*}& & \text{logit} \left( {\pi }_{i} \right) ={\alpha }_{1}\times {\mathrm{PercentTree}}_{i}+{\alpha }_{2}\times {\mathrm{PercentLawn}}_{i}+{\alpha }_{3}\times {\mathrm{GlassArea}}_{i}\end{eqnarray*}

where *α*_1_ is the increase in the log-odds of an excess zero for each percent increase in tree area, *α*_2_ is the change in the log-odds for each percentage increase in lawn area, and *α*_3_ is the increase in log-odds associated for each 175.63 m^2^ of glass area on a building.

Prior distributions were required to complete the Bayesian hierarchical model. All parameters for both the Poisson and binomial model were given non-informative normal priors centered at 0 with variance 10 and the temporal term *δ*_*t*_ followed a random walk prior of order one. This prior allows each *δ*_*t*_ term to be correlated with the previous *δ*_*t*−1_ term with variance 1,000 and assumes that fatal collisions are more associated in consecutive months than collisions in non-consecutive months.

We estimated the model parameters using Markov chain Monte Carlo (MCMC) in WinBUGS (Spiegelhalter et al., 2002) (Supplemental Information). We ran one MCMC chain for 30,000 iterations with a burn-in of 15,000. The remaining sample was thinned to every 3 iterations, yielding a final posterior sample of size 5,000 for computing posterior summaries. We assessed convergence through visual inspection of trace and density plots for each parameter and Geweke’s diagnostic (Geweke, 1992). For parameter estimates, we sampled from the joint posterior distribution and report the mean posterior estimates and the 95% credible intervals on the relative risk scale. We also plotted the estimated relative risks for each building month to month.

## Results

We documented a total of 240 collision deaths representing 55 identifiable species at the VTCRC during the course of the study (Table 1). Forty-one (17%) of the bird deaths were unidentifiable remains. The average number of surveys per building for the study period was 136. All 22 buildings surveyed had at least one recorded collision death. Of the 298 total survey days, 47 occurred on days in 2013, 198 days in 2014, and 53 in 2015. We conducted 92 surveys in the fall, 75 in the winter, 96 in the spring, and 35 in the summer.

**Table 1:** Bird-window collision numbers. Species observed as window collision deaths at the VTCRC (October 2013–May 2015).

Family	Common name	Scientific name	Fall (9/1-11/30)	Winter (12/1-2/28)	Spring (3/1-5/31)	Summer (6/1-8/31)	Total collisions
Charadriidae	Killdeer	*Charadrius vociferus*	–	–	1	–	1
Scolopacidae	American Woodcock^a^	*Scolopax minor*	–	1	–	–	1
Columbidae	Mourning Dove	*Zenaida macroura*	5	2	4	3	14
Cuculidae	Yellow-billed Cuckoo^a^	*Coccyzus americanus*	1	–	4	–	5
Caprimulgidae	Common Nighthawk	*Chordeiles minor*	1	–	–	–	1
Trochilidae	Ruby-throated Hummingbird	*Archilochus colubris*	15	–	7	2	24
Tyrannidae	Great Crested Flycatcher	*Myiarchus crinitus*	–	–	–	1	1
Tyrannidae	Eastern Kingbird^a^	*Tyrannus tyrannus*	–	–	2	–	2
Picidae	Northern Flicker	*Colaptes auratus*	–	–	2	2	4
Picidae	Yellow-bellied Sapsucker	*Sphyrapicus varius*	1	1	–	–	2
Picidae	Unknown Woodpecker	*Woodpecker spp.*	1	–	–	–	1
Vireonidae	Red-eyed Vireo	*Vireo olivaceus*	1	–	–	–	1
Vireonidae	Blue-headed Vireo	*Vireo solitarius*	1	–	–	–	1
Vireonidae	Yellow-throated Vireo	*Vireo flavifrons*	–	–	–	1	1
Paridae	Carolina Chickadee	*Poecile carolinensis*	1	–	–	–	1
Paridae	Tufted Titmouse	*Baeolophus bicolor*	1	–	–	–	1
Sittidae	White-breasted Nuthatch	*Sitta carolinensis*	1	–	–	–	1
Certhiidae	Brown Creeper	*Certhia americana*	1	–	–	–	1
Regulidae	Golden-crowned Kinglet	*Regulus satrapa*	7	2	–	–	9
Regulidae	Ruby-crowned Kinglet	*Regulus calendula*	–	1	1	–	2
Turdidae	American Robin	*Turdus migratorius*	2	–	11	8	21
Turdidae	Hermit Thrush	*Catharus guttatus*	3	–	1	–	4
Turdidae	Unknown Thrush	*Thrush spp.*	1	–	–	–	1
Turdidae	Eastern Bluebird	*Sialia sialis*	–	–	2	–	2
Mimidae	Brown Thrasher	*Toxostoma rufum*	–	–	2	–	2
Mimidae	Gray Catbird^a^	*Dumetella carolinensis*	2	–	10	3	15
Mimidae	Northern Mockingbird	*Mimus polyglottos*	1	–	–	1	2
Sturnidae	European Starling	*Sturnus vulgaris*	–	–	2	–	2
Motacillidae	American Pipit	*Anthus rubescens*	–	1	–	–	1
Bombycillidae	Cedar Waxwing	*Bombycilla cedrorum*	2	–	3	2	7
Parulidae	American Redstart	*Setophaga ruticilla*	–	–	1	–	1
Parulidae	Black-throated Blue Warbler	*Setophaga caerulescens*	1	–	–	–	1
Parulidae	Black-throated Green Warbler	*Setophaga virens*	2	–	–	–	2
Parulidae	Chestnut-sided Warbler	*Setophaga pensylvanica*	1	–	–	–	1
Parulidae	Common Yellowthroat	*Geothlypis trichas*	2	–	–	1	3
Parulidae	Ovenbird	*Seiurus aurocapilla*	–	–	–	3	3
Parulidae	Yellow-rumped Warbler	*Setophaga coronata*	–	–	1	–	1
Parulidae	Louisiana Waterthrush	*Parkesia motacilla*	–	–	–	1	1
Parulidae	Northern Waterthrush	*Parkesia noveboracensis*	1	–	–	–	1
Parulidae	Palm Warbler	*Setophaga palmarum*	1	–	–	–	1
Parulidae	Tennessee Warbler	*Oreothlypis peregrina*	1	–	–	–	1
Parulidae	Worm-eating Warbler	*Helmitheros vermivorum*	–	–	1	–	1
Parulidae	Yellow Warbler	*Setophaga petechia*	1	–	–	–	1
Emberizidae	Dark-eyed Junco	*Junco hyemalis*	3	2	4	–	9
Emberizidae	Eastern Towhee^a^	*Pipilo erythrophthalmus*	1	1	–	–	2
Emberizidae	Field Sparrow^a^	*Spizella pusilla*	–	–	–	1	1
Emberizidae	Fox Sparrow	*Passerella iliaca*	1	–	–	–	1
Emberizidae	Song Sparrow	*Melospiza melodia*	1	–	2	3	6
Emberizidae	White-throated Sparrow	*Zonotrichia albicollis*	5	2	2	–	9
Emberizidae	White-crowned Sparrow	*Zonotrichia leucophrys*	–	1	–	–	1
Emberizidae	Savannah Sparrow	*Passerculus sandwichensis*	–	–	1	–	1
Cardinalidae	Indigo Bunting	*Passerina cyanea*	1	–	2	–	3
Cardinalidae	Northern Cardinal	*Cardinalis cardinalis*	1	1	1	2	5
Cardinalidae	Scarlet Tanager	*Piranga olivacea*	1	–	–	–	1
Icteridae	Red-winged Blackbird	*Agelaius phoeniceus*	–	–	1	2	3
Fringillidae	American Goldfinch	*Spinus tristis*	1	–	4	–	5
Fringillidae	House Finch	*Haemorhous mexicanus*	1	–	–	2	3
	Unknown	*Unidentified*	16	2	11	12	41
		*TOTAL*	90	17	83	50	240

**Notes.**

a Virginia Species of Greatest Conservation Need (VDGIF 2015).

**Figure 3:**
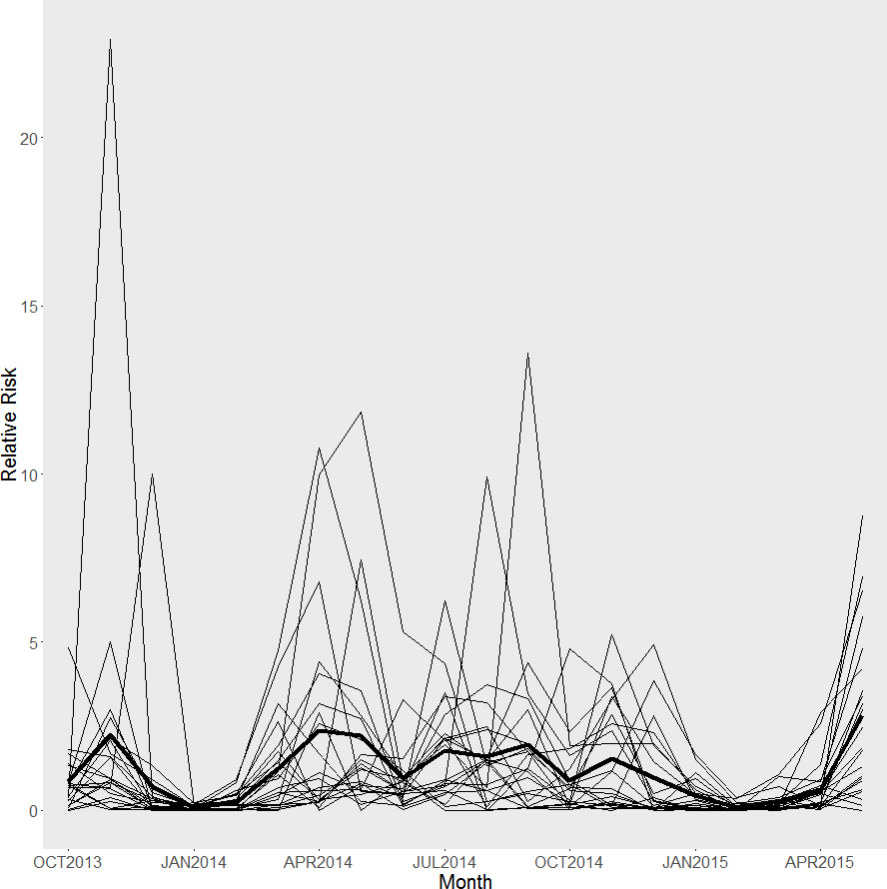
Relative risk. Estimated relative risk of fatal collisions for each building at each month. The thicker black line represents the average relative risk across time.

Our model converged based on visual inspection of trace and density plots for the parameters and according to Geweke’s Diagnostics (Table 2). Overall, we found significant effects for percent lawn area within 50 m of a building and glass area on a building. Specifically, we found a significantly increased relative risk of 1.30 (95% CRI [1.05–1.65]) for fatal collisions for each 175.6 m^2^ of additional glass area on a building; and a significantly reduced risk of 0.96 (95% CI [0.93–0.98]) for collisions for each percent increase in lawn area within 50 m of a building. We also found a marginally significant relationship between collisions and the percent area of trees within 50 m of a building. However, we found no significant relationships between the variables and the log-odds of no fatal collision (the excess zeroes in the model). We also observed decreased relative risk of collisions between December and March when we plotted the relative risks (Fig. 3).

### Glass area and surrounding land cover

**Figure 4:**
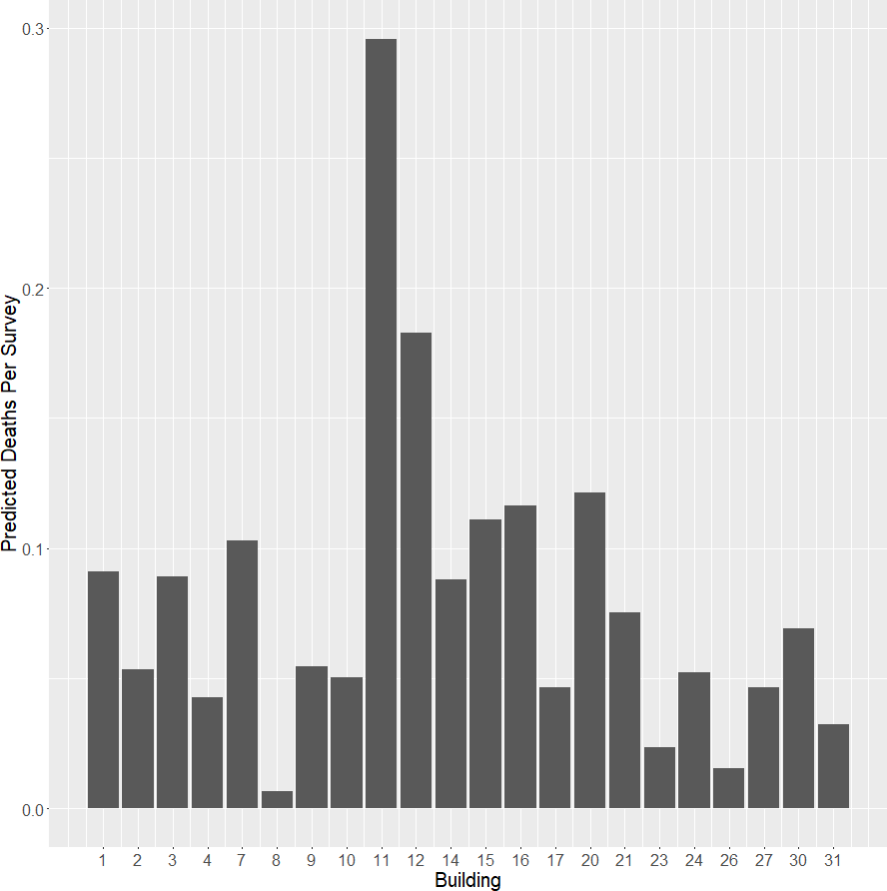
Collisions by building. Mean number of collision deaths per survey for each building surveyed at the VTCRC (October 2013–May 2015).

Seven buildings were responsible for the majority (57.1%) of window collision deaths (Fig. 4). Buildings 11 and 12 had the highest avian mortality and accounted for a quarter (24.6%) of all documented collision deaths. Although we were unable to obtain window specifications on all buildings to make quantitative comparisons of reflectivity and glass type, observations from our surveys suggest that window reflectivity plays a large role in the frequency of collisions at the VTCRC. The amount of glass on buildings ranges from 18 m^2^ (building 8) to 693 m^2^ (building 9). Based on our model, this feature played a significant role in fatal collisions. Building 8, with the least amount of glass, had only one recorded collision death. The two buildings that killed the most birds, 11 and 12, have an estimated 451 m^2^ and 492 m^2^ of glass, respectively. Buildings 1 and 2 are almost identical buildings located adjacent to each other. Building 1, with 219 m^2^ glass area, killed 15 birds; building 2, with 191 m^2^ glass area, killed nine. A notable difference between the two buildings is the amount of forest area (840 m^2^) on one side of building 1, while building 2 has no surrounding forest area.

**Table 2:** Relative risk. Posterior relative risk and log-odds estimates for fatal collisions. The *β* parameters are on the relative risk scale, and the *α* parameters are on the log-odds scale. A parameter is considered significant if the credible interval does not contain 1. Geweke’s Diagnostic should be between −1.96 and 1.96 for a parameter to be considered converged.

Effect	Posterior mean	95% credible interval	Geweke’s diagnostic
PercentTree, *β*_1_	1.02	1.00, 1.05	0.23
PercentLawn, *β*_2_	0.96	0.93, 0.98	−0.53
GlassArea, *β*_3_	1.30	1.05, 1.65	−1.29
PercentTree, *α*_1_	0.99	0.95, 1.03	−0.54
PercentLawn, *α*_2_	0.99	0.97, 1.00	0.53
GlassArea, *α*_3_	0.85	0.56, 1.32	−1.48

Impervious area (51%) consisting of parking lots, sidewalks, and roads dominate the surrounding 50 m of all buildings in the VTCRC. Lawn (35%), tree (10%), forest (3%), and open water (1%) comprise the remaining area. Our model indicated that lawn area mitigated the relative risk of fatal collisions while tree area was associated with a slight increase in risk of collision. Building 11 has the highest percentage of surrounding forest cover (25.2%), while building 12 has no surrounding forest area. Building 11 also has the least amount of lawn area compared to the other buildings.

### Seasonality

**Figure 5:**
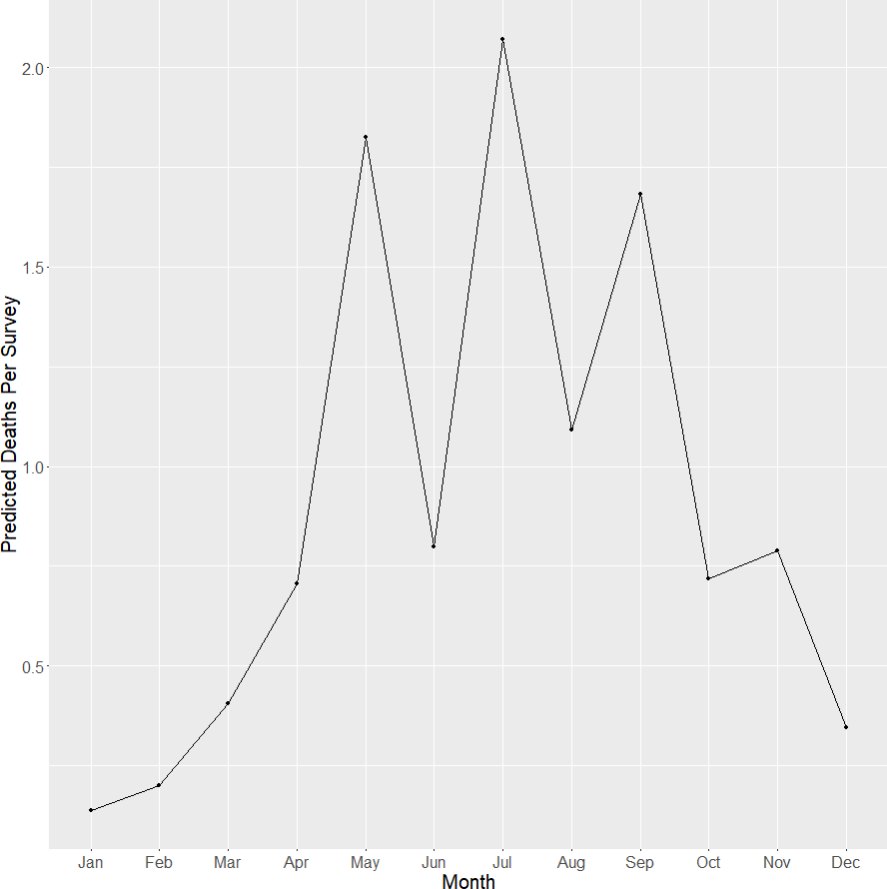
Collisions by month. Mean number of collision deaths per survey for each month at the VTCRC (October 2013–May 2015). Months were combined across years.

July, May, and September had the highest mortality respectively when correcting for uneven survey effort (Fig. 5). Collision deaths were lowest in the winter months. The species with the most collision deaths in the fall were Ruby-throated Hummingbird (*n* = 15), Golden-crowned Kinglet (*Regulus satrapa)* (*n* = 7), Mourning Dove (*Zenaida macroura*) (*n* = 5), and White-throated Sparrow (*n* = 5). In the winter, White-throated Sparrow (*n* = 2), Mourning Dove (*n* = 2), Golden-crowned Kinglet (*n* = 2), and Dark-eyed Junco (*Junco hyemalis*) (*n* = 2) were the most frequent colliders. American Robin (*Turdus migratorius*) (*n* = 11), Gray Catbird (*Dumetella carolinensis*) (*n* = 10), and Ruby-throated Hummingbird (*n* = 7) dominated in the spring. In the summer, American Robin (*n* = 8), Mourning Dove (*n* = 3), Gray Catbird (*n* = 3), Ovenbird (*n* = 3), and Song Sparrow (*Melospiza melodia*) (*n* = 3) were most frequently detected.

### Species

The Ruby-throated Hummingbird was the most common window collision species and accounted for 10% of all deaths recorded during our entire study. The majority (29.2%) of hummingbird deaths were at building 11. The next three most common species, American Robin, Gray Catbird, and Mourning Dove, accounted for 21% of identifiable deaths combined. Nine of the 55 species we documented are disproportionately vulnerable to collisions at all building types (Loss et al., 2014). Six Virginia Species of Greatest Conservation Need (Virginia Department of Game and Inland Fisheries, 2015) were documented as window kills, including American Woodcock (*Scolopax minor*), Eastern Towhee (*Pipilo erythrophthalmus*), and Field Sparrow (*Spizella pusilla*) (Table 1).

The most common families were Emberizidae (13% of collision deaths), Turdidae (12%), Trochilidae (10%), Mimidae (8%) and Parulidae (8%). Emberizidae collisions were mostly Dark-eyed Junco (*n* = 9) and White-throated Sparrow (*n* = 9). American Robin (*n* = 21) represented the majority of Turdidae collisions. Ruby-throated Hummingbird was the only species within Trochilidae. Within Mimidae, Gray Catbird (*n* = 15) was the most common species. Thirteen species comprised Parulidae, with Common Yellowthroat (*Geothlypis trichas*) and Ovenbird (*n* = 3) comprising most of the collisions.

## Discussion

Our documentation of 240 birds over 20 months suggest that avian fatalities are a significant problem at the VTCRC. We found collision victims year-round because the reflective windows did not present a barrier to avoid. Results of our study indicate a positive correlation between the number of fatal collisions and glass area. Glass or window area is one of the primary factors affecting collisions as previous studies have shown (Klem et al., 2009; Borden et al., 2010; Hager et al., 2013; Cusa, Jackson & Mesure, 2015; Ocampo-Peñuela et al., 2016) and our findings provide additional evidence for this. In addition to glass area, we speculate that collision frequency at the VTCRC buildings is related to the configuration of glass on building façades. Several buildings (e.g., 12, 11, and 7) contain a large portion of glass concentrated in one area. Additionally, many of the buildings in this office park have darkly tinted, though nonetheless reflective, windows. The buildings that experienced a high number of collisions, including building 11, exhibit a mirror-like reflection of the immediate environment that can confuse birds by projecting a false image of habitat. Building 11 also had the highest amount of surrounding forest cover, a factor that may have contributed to a higher number of collisions. Measuring the degree of reflectivity, a trait that can vary by type of glass, cloud cover, angle of the sun, time of day, and by façade, proved to be beyond the scope of our study.

In our analyses of land cover characteristics, lawn area was negatively correlated with the number of bird fatalities in both our exploratory linear regression and the reported Bayesian model. This finding may be attributed to these areas attracting fewer birds than more heavily vegetated, resource-laden areas in the vicinity. Small sample size limited our ability to fully explore the relationship between nearby vegetation and the number of fatal collisions. Of the 22 buildings surveyed, only six had forest cover within 50 m; therefore, we were unable to quantify possible effects of this variable. While the amount of impervious surface in the vicinity of buildings was not significantly related to collisions, ornamental trees were found to be marginally significant to the number of collisions we recorded. Our analyses did not take into account potential effects of variables such as building size, window reflectivity, nocturnal lighting, and local avian abundance, all of which would be pertinent focuses of future research at this site (Wittig et al., 2017).

Our results indicate that birds strike windows year-round at the VTCRC. Mitigating efforts should be implemented across the breeding season and both spring and fall migration to be most effective. Although we documented the highest number of fatalities during May and September, July had the most bird deaths per survey. The number of collisions in July is notable because the majority of related studies focus survey efforts during the spring and fall migration and little research is conducted during the breeding season (O’Connell, 2001; Gelb & Delacretaz, 2006; Hager et al., 2008; Klem et al., 2009; Borden et al., 2010; Bayne, Scobie & Rawson-Clark, 2012; Loss et al., 2014). Bird deaths in July are likely due to the number of newly fledged birds that are unfamiliar with the surrounding landscape (Hager, 2014; Kahle, Flannery & Dumbacher, 2016). Similar to Hager & Craig (2014), the species most frequently detected during summer was the American Robin.

Consistent with other studies showing their tendency to be frequent colliders (Klem, 1989; Hager & Craig, 2014; Loss et al., 2014), we found that Ruby-throated Hummingbirds fatally struck windows more often than any other species. Hummingbirds may be at increased risk of collision compared with other species because of their small size and delicate stature, as well as their high flight velocities (Kahle, Flannery & Dumbacher, 2016). This combination could make them less likely to survive an impact with a solid barrier. Additional research is needed to assess how relative species size and robustness correlate with strike susceptibility in hummingbirds and other species. Our results support the finding that migrants are especially prone to collisions, but suggests that resident species are also at risk. Notably, six of the species we documented are listed as Virginia Species of Greatest Conservation Need (Virginia Department of Game and Inland Fisheries, 2015), a designation signifying their decline in the state and the need for conservation action.

The actual number of bird-window collision deaths is likely higher than documented at the VTCRC (O’Connell, 2001). Predators and scavengers undoubtedly played a role in the detection of carcasses (Hager, Cosentino & McKay, 2012; Kummer et al., 2016), especially since the time interval between surveys varied. Evidence of raccoons (e.g., scat piles) was common on survey routes and the VTCRC managed a feral cat colony at the time of this study. Ground crews hindered detectability when mowing and leaf collection occurred. We did not account for searcher detection error, and we acknowledge that some carcasses likely went undetected during our study (Smallwood, 2007; Parkins, Elbin & Barnes, 2015). Detectability at buildings varied due to surrounding substrate; building 9, which contains the highest amount of glass, also has heavy ivy groundcover along the building perimeter that likely contributed to lower detectability.

Mitigation measures that appear most effective are window films or netting applied to the outside of windows (Klem, 1990; Ocampo-Peñuela et al., 2016). Due to the highly reflective quality of the windows in the VTCRC, collision deterrents must be applied to window exteriors in order to increase glass visibility. An efficient approach would target buildings shown to be most hazardous to birds, including buildings 11, 12, 20, 16, and 15. Closing interior blinds has little to no effect on the external appearance of the glass at many VTCRC windows. Due to the press this project received (Supplemental Information), we made contact with several architects to suggest that plans for VTCRC Phase II incorporate bird-window collision mitigation measures and less reflective glass. Options for new construction at the VTCRC include using less reflective glass, fritted glass, smaller windowpanes, and angling windows upon installation so they do not reflect the sky or adjacent vegetation (Klem et al., 2004).

VTCRC management can reduce the number of birds that die each year by retrofitting the most hazardous buildings to increase visibility to birds. Although we identified specific “problem” buildings that experienced a higher number of collisions, ultimately management did not grant permission when we requested to field-test collision deterrents. The reasons given for not altering windows included general aesthetics, potential damage to windows, interference with heating and cooling systems, policy, and cost. Our communications with VTCRC management ended when they stated they would follow Virginia Tech policy addressing bird-window collisions. Coincidentally, there is no Virginia Tech policy addressing avian collisions. A number of Virginia Tech offices on campus were cooperative in investigating bird-window collisions, and we conducted a small pilot study on the university campus from fall 2014 to spring of 2015 (Supplemental Information). When we requested to test mitigation measures at one building, Fralin Biotechnology Center, we were not permitted due to aesthetic reasons.

We must consider the cumulative impacts of bird-window collisions nationwide. As defined by the National Environmental Protection Act (1969), “Cumulative impacts can result from individually minor but collectively significant actions taking place over a period of time”. In the near future, we hope studies that ignite mitigation actions will become more commonplace, as with those implemented at Duke University (Ocampo-Peñuela et al., 2016). College campuses can include bird-safe buildings as a component of their sustainability campaigns. Bird-window collision studies performed on campuses provide students with an opportunity to investigate human impacts on the natural world where they live, work, and study.

While we acknowledge the overwhelming scope of bird-window collisions (Loss et al., 2014; Machtans & Thogmartin, 2014), we view the findings of our research as a call to action. To protect avian life at the VTCRC and the Virginia Tech campus in the future, testing and implementing permissible mitigating strategies should be an important component of bird-window collision research. Applying mitigation measures such as window films to the few buildings with high avian mortality would be a reasonable approach. Ultimately, a University policy on minimizing bird-window collisions would help guide future construction, encourage modification to buildings known to be particularly threatening to birds, and make the school a leader in sustainable, bird-friendly building.

##  Supplemental Information

10.7717/peerj.4562/supp-1 Supplemental Information 1 VTCRC collision data 2013–2015 Raw data.

10.7717/peerj.4562/supp-2 Supplemental Information 2 This file contains code to manage and analyze the collected data according to the methods outlined in our manuscript; data necessary to run this code can be accessed in additional supplementary files, Deaths.csv, Windows.csv, and VTCRC_Collisions.csv Raw data.

10.7717/peerj.4562/supp-3 Supplemental Information 3 This file contains code to set up data and implement the MCMC used for Bayesian inference in our manuscript; data necessary to run this code can be accessed in additional supplementary files, Deaths.csv, Windows.csv, and VTCRC_Collisions.csv Raw data.

10.7717/peerj.4562/supp-4 Supplemental Information 4 This file contains data on the number of fatal bird collisions per building Raw data.

10.7717/peerj.4562/supp-5 Supplemental Information 5 This file contains data on the number of facades per building Raw data.

10.7717/peerj.4562/supp-6 Supplemental Information 6 This file contains data on the measured covariates recorded for each building Raw data.

10.7717/peerj.4562/supp-7 Supplemental Information 7 Press regarding bird-window collisions at the Virginia Tech Corporate Research Center

10.7717/peerj.4562/supp-8 Supplemental Information 8 Raw data from campus pilot study Official survey and incidental reports of bird-window collisions on the Virginia Tech campus (September 2014–May 2015).

## Additional Information and Declarations

### Data Availability

The following information was supplied regarding data availability: The raw data have been provided as Supplemental Files and here: Zirkle, Keith; rmurray@vt.edu (2018): Files for Schneider et al. (2018). Figshare. https://doi.org/10.6084/m9.figshare.5982394.v1.
