# Supplementary material for: Year-round monitoring reveals prevalence of fatal bird-window collisions at the Virginia Tech Corporate Research Center
Source: PeerJ. 2018 Apr 4;6:e4562. doi: 10.7717/peerj.4562 (PMC5889704; doi:10.7717/peerj.4562)
Supplement: Supplemental Information 7 [file peerj-06-4562-s007.docx]

Press regarding bird-window collisions at the Virginia Tech Corporate Research Center.

| **Date** | **Media** | **Title** | **Link** |
| --- | --- | --- | --- |
| 12/26/2013 | WVTF | Hope Has Wings | <http://wvtf.org/post/hope-has-wings> |
| 2/9/2014 | Natural Awakenings magazine | Virginia Tech Group Works to Protect Birds | <https://issuu.com/nablueridge/docs/february_2014> |
| 11/11/2014 | WVTF | Volunteer Researchers Work to Prevent Bird Collisions | <http://wvtf.org/post/volunteer-researchers-work-prevent-bird-collisions#stream/0> |
| 11/17/2014 | Virginia Tech News | Birds and windows -- a deadly dilemma | <http://www.vtnews.vt.edu/articles/2014/11/111714-cnre-birdwindowcollisions.html> |
| 11/21/2014 | WSLS 10 | Virginia Tech researcher works to protect birds from windows | <http://wsls.com/2014/11/21/virginia-tech-researcher-works-to-protect-birds-from-windows/> |
|  | Project Blog | Hope is the thing with feathers | <http://birds-and-windows.blogspot.com> |
